# Specialized medical care in primary care using telemedicine in Northeast Brazil: a descriptive study, Rio Grande do Norte, Brazil, 2022-2023

**DOI:** 10.1590/S2237-96222025v34e20240256.en

**Published:** 2025-03-14

**Authors:** Maria Eulália Vinadé Chagas, Gabriel Ricardo Fernandes, Deysi Heck Fernandes, Andressa Dutra Dode, Gabriela Tizianel Aguilar, Tiago Sigal Linhares, Marcilene Batista Costa, Haylla Travassos Caires, Felipe Cezar Cabral, Hilda Maria Rodrigues Moleda Constant, Taís de Campos Moreira

**Affiliations:** 1 Hospital Moinhos de Vento, Responsabilidade Social, Porto Alegre, RS, Brasil; 2 Universidade do Vale do Rio dos Sinos, Escola de Saúde, São Leopoldo, RS, Brasil; 3 Universitat de València, Department of Behavioral Sciences Methodology, Valencia, Comunidad Valenciana, Espanha

**Keywords:** Telemedicine, Noncommunicable Diseases, Equity, Primary Health Care, Epidemiology, Descriptive, Telemedicina, Enfermedades no Transmisibles, Equidad, Atención Primaria de Salud, Epidemiología Descriptiva

## Abstract

**Objective:**

To describe an interconsultation project with specialists and family health teams participating in a telemedicine project in the state of Rio Grande do Norte, Brazil.

**Methods:**

Descriptive study with evaluation of TeleNordeste Project interconsultations. Data collection began in November 2022, with consultations with a cardiologist, neurologist, psychiatrist and endocrinologist made available to primary health centers. Patients over 18 years of age were included in the study. Quantitative data were described as medians and percentiles, qualitative data were measured in absolute frequency and percentage.

**Results:**

572 patients were included and carried out 847 consultations; 71% were women, the median age were 50 years, 96.7% of patients had chronic non-communicable diseases. The median of the waiting time for consultation was 7 days. In total, 565 patients had their complaints completely resolved and did not need to be referred to a specialized service.

**Conclusion:**

The TeleNordeste Project brought to Rio Grande do Norte a type of medical care facilitated by digital health, with the possibility of agile contact and easy access in primary health care to cardiologists, neurologists, psychiatrists and endocrinologists, enabling improved care and increased effectiveness in real time.

## Resumen

**Objetivo:**

Describir un proyecto de interconsulta con especialistas y equipos de salud de la familia participantes de un proyecto de telemedicina en el estado de Rio Grande do Norte, Brasil.

**Métodos:**

Estudio descriptivo con evaluación de las interconsultas del Proyecto TeleNordeste. La recolección de datos comenzó en noviembre de 2022, poniendo a disposición de los centros de atención primaria de salud las consultas con cardiólogo, neurólogo, psiquiatra y endocrinólogo. Se incluyeron en el estudio pacientes mayores de 18 años. Los datos cuantitativos se describieron como medianas y percentiles, los datos cualitativos se midieron en frecuencia absoluta y porcentaje.

**Resultados:**

Se incluyeron 572 pacientes y se realizaron 847 consultas; el 71% eran mujeres con una mediana de edad de 50 años; el 96,7% de los pacientes presentaban enfermedades crónicas no transmisibles. La mediana del tiempo de espera para consulta fue de 7 días. En total, 565 pacientes tuvieron sus quejas completamente resueltas y no necesitaron ser derivados a un servicio especializado.

**Conclusión:**

El Proyecto TeleNordeste trajo a Rio Grande do Norte un tipo de atención médica facilitada por la salud digital, con posibilidad de contacto ágil y fácil acceso a cardiólogos, neurólogos, psiquiatras y endocrinólogos en la atención primaria de salud, permitiendo mejorar la atención y aumentar la efectividad en tiempo real.

**Ethical aspects**

**This research respected ethical principles, having obtained the following approval data:**

Research ethics committee: Hospital Moinhos de Vento

Opinion number: 5,744,857

Approval date: 11/8/2022

Certificate of submission for ethical appraisal: 63070522.8.0000.5330

Consent form: Obtained from all participants before telemedicine consultation.

## Introduction

Adoption of information technologies in health has expanded the diversification of telemedicine, encompassing not only medical assistance, but also e-learning and teleconsultations, such as second opinions given by experts ^1^ ^,^ ^2^. Digital health provides access that is equivalent to access to care available in urban centers when it occurs synchronously ^1^ ^,^ ^2^, and is important for achieving the targets of the third Sustainable Objective of the 2030 Agenda, which focuses on universal coverage as a necessary path to health and well-being, from an economic point of view and from a perspective of safe and effective access to care, medicines and vaccines ^2^ ^,^ ^3^.

In Brazil, health inequities are influenced both by territorial extension and by the distribution of the density of doctors ^4^. The Northeast has 1.93 doctors per 1,000 inhabitants, being the second region with one of the lowest densities in the country. The multiple realities of access to specialized healthcare create care gaps, which are intensified by the lack of horizontal management and care coordination aligned at the three levels of management: municipal, state and federal ^5^ ^-^ ^7^. Irregular health coverage with doctors is a greater concern in areas where the human development index is very low, such as in Northeast Brazil ^8^. This inequity, inherited from limitations of explicit regional policies in the health system ^9^ and the disjointed distribution of funding transfers, highlights the premise of the inverse care law ^10^, due to availability of medical care being inverse to social needs ^11^. This trend reinforces the importance of rethinking the organization of health services outside the logic of the market economy ^9^ ^,^ ^12^.

Understanding the challenges in access to healthcare, including the unequal distribution of doctors and the scarcity of human resources for administrative tasks, highlights the urgency of projects that promote improvement of the Brazilian National Health System (*Sistema Único de Saúde* - SUS) and meet the specific demands of each region of the country, especially in the area of primary health care. In this way, through a public-private partnership, the SUS Institutional Development Support Program, in line with the Global Strategy on Digital Health 2020-2025, has proposed, with five hospitals of excellence in Brazil, jointly with the Ministry of Health and the National Council of Health Secretaries, the eveloppment of the project entitled “Specialized Medical Care in the Northeast Region of Brazil using Telemedicine - TeleNordeste Project.” The purpose of the project is to provide assistance and support to health service management in the Northeast region of the country. The objective of this study is to describe an interconsultation project with specialists and family health teams participating in a telemedicine project in the state of Rio Grande do Norte, Brazil.

## Methods

### Design

This is a descriptive study of interconsultations that took place via the TeleNordeste project between 11/30/2022 and 11/30/2023, analyzing the demographic characteristics, chronic conditions of the patients treated and the monitoring of referrals made for face-to-face care. Data were reported in accordance with guidelines for cross-sectional studies ^13^.

*Background*

TeleNordeste is a health care and research project, developed within the scope of the SUS Institutional Development Support Program, which began in November 2022 and aims to offer consultations with a cardiologist, neurologist, psychiatrist and endocrinologist for primary health centers. The specialty doctors were located in the state of Rio Grande do Sul, in the southern region of Brazil. The primary care centers were located in three health regions of the two macro-regions of the state of Rio Grande do Norte, located in Northeast Brazil. A telemedicine platform developed by the TeleNordeste project was used, through which patient registration, appointments, consultations were carried out and through which the specialist recorded the patient’s progress. To guarantee the security of the platform and the confidentiality of the consultation, only the TeleNordeste project team and the Family Health Team doctors and nurses had access to the tool, with personal usernames, received after training on the use of the platform and confirmation of the terms of acceptance of use and the platform’s privacy policies. The telemedicine platform underwent evaluation by external agents to verify the security mechanisms and was approved for use. There was no participant video or voice storage in the consultations. All participant personal data in the interconsultations were stored on the platform in accordance with the criteria stipulated by the General Personal Data Protection Law ^14^. 

The consultations were carried out via videoconference, in a triangulated manner between the specialist doctor, the primary health care doctor and the patient, in the form of interconsultation, that is, the exchange of information and opinions between doctors for diagnostic investigation or therapeutic and clinical management ^15^. Care triangulation aims to strengthen the SUS decentralization principle and the attributes of primary health care, with the presence of the local territory doctor, thus maintaining the coordination, completeness and longitudinality of care. 

### Participants

Patients over 18 years of age with demands for cardiology, endocrinology, psychiatry or neurology medical specialties were eligible for interconsultation in the TeleNordeste project and included in the research by means of convenience sampling.

**Figura 1.**
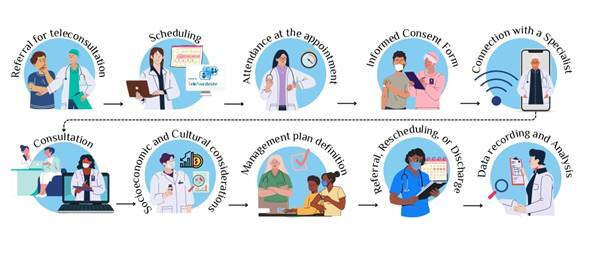
Patient process in the TeleNordeste Project right from indication of interconsultation through to definition of procedure and analysis of patient data

The consultation date was determined by the primary health care medical professional’s appointment diary. On the scheduled day and time, the patient attended the primary care center, the free and informed consent form was explained and signed and then, together with their referring doctor, they connected with the specialist. The consultation was conducted with the three parties actively interacting, with the specialist suggesting actions or requesting additional information to understand the cases completely. If necessary, the focus specialist doctor could ask the primary care center doctor to perform a physical examination, or specific medical maneuvers, offering guidance on how to perform them, as well as requesting them to provide more details of the anamnesis information. At the end of the interconsultation, the patient could be discharged, have an appointment made for a new consultation for continuing care or be referred for in-person care. All care plan procedures were defined jointly by the doctors, taking into account the patient’s socioeconomic and cultural aspects, in addition to the resources available via SUS in the region (Figure 1).

### Variables

The primary outcome was effective care, considered to be when the main complaint was resolved during interconsultation. The study variables included sex, age, presence of chronic diseases, multimorbidity, use of the appointment diary and group of chronic noncommunicable diseases.

### Data source and measurement

The study data were collected between November 2022 and November 2023, by consultating the patients’ medical records, and stored on electronic spreadsheets. Only patients who signed a free and informed consent form were included.

Chronic non-communicable diseases were categorized into 12 groups, according to the Global Burden of Disease, with the exception of the additional group entitled “other metabolic diseases”, which was prepared by the authors ^16^. This group classified patients who had a metabolic syndrome unrelated to diabetes or kidney problems, given the high prevalence of the condition in patients treated. 

### Statistical methods

We used descriptive statistics, with calculation of medians and percentiles for continuous data and absolute frequency and percentage for categorical data.

## Results

In all, 572 patients were included in the study. They had 847 consultations via the TeleNordeste project. The median age of patients treated was 50 years (interquartile range 36;62). Their demographic characteristics are shown in Table 1.

**Table 1:** Demographic characteristics of patients receiving care via the TeleNordeste Project. Rio Grande do Norte, Brazil, 2022-2023 (n=572)

Variable	n (%)
**Sex**	572 (100)
Female	406 (71)
Male	166 (29)
**Chronic noncommunicable disease**	553 (96.7)
**Multimorbidity**	553 (96.7)

Care was considered effective for 98.7% (n=565) of patients who had their main complaint resolved during the interconsultations, without the need for referral for in-person care in the same specialty. Among the seven patients who needed to be referred for face-to-face care, three were referred to cardiology services, two to endocrinology services, one to psychiatry services and one to neurology services. The reasons for referral were as follows: cardiology: one for heart surgery and two with preoperative reports showing risk regarding heart surgery; endocrinology: one with type 1 diabetes and one with macrovascular complications; psychiatry: patient referred for emergency assessment; neurology: patient referred for assessment regarding administration of botulinum toxin.

Of the 847 consultations, 71.8% (n=608) were for first interconsultations and 28.2% (n=239) for continuing care. Median waiting time for consultation taking the four specialties as a whole was 7 days. Median waiting time for cardiology was 5 days; 7 days for psychiatry and neurology; and 10 days for endocrinology.

Use of appointment agendas per specialty in the project, taken as the ratio between the number of completed interconsultations and the number of interconsultations with appointments, can be seen in Table 2.

**Table 2:** Use of appointment agenda according to specialty. Rio Grande do Norte, Brazil, 2022-2023 (n=572)

Specialty	Use of appointment agenda (%)
Cardiology	235 (84)
Endocrinology	281 (75)
Neurology	117 (80)
Psychiatry	214 (84)

Data were collected on the main chronic noncommunicable diseases of the 572 patients treated. This resulted in 1,149 reported diseases, which were classified into 12 groups, as shown in Table 3.

**Table 3:** Absolute number and frequency of the main chronic noncommunicable diseases. Rio Grande do Norte, Brazil, 2022-2023 (n=1.149)

Disease group	n (%)
Cardiovascular diseases	364 (60,5)
Diabetes and kidney disease	293 (51.2)
Other metabolic diseases	182 (31.8)
Mental disorders	157 (27.5)
Neurological disorders	77 (13.5)
Musculoskeletal disorders	25 (4.4)
Substance use disorders	20 (3.5)
Sense organ diseases	7 (1.2)
Chronic respiratory diseases	6 (1.0)
Digestive diseases	5 (0,9)
Neoplasms	4 (0.7)
Other^a^	9 (1.6)

Note: ^a^Anemia, chronic pain, gout, lupus, uterine fibroids and poliomyelitis.

## Discussion

Implementation of the TeleNordeste project brought to Rio Grande do Norte a type of medical care facilitated by digital health, with the possibility of agile contact and easy primary health care access to cardiologists, neurologists, psychiatrists and endocrinologists, enabling the improvement of care and increased effectiveness in real time. For this project, measurement of the effectiveness of care was defined as the ability to resolve a complaint without the need to refer the patient to another level of care. High effectiveness was achieved, since 565 patients treated during the period had their complaints completely resolved and did not need to be referred to a specialized service.

In Brazil, it is estimated that primary health care is capable of solving around 80% of the population’s health problems, and only 4% to 9% of patients treated are referred to specialized levels of care ^17^ ^,^ ^18^. It has been found that, in most cases, referrals from primary care to specialized care services are, to a large extent, determined by the doctor’s previous experience in managing the disease in question, so that the demand for referrals also varies depending on the specialty or expertise of the doctor who works in primary care ^19^. The TeleNordeste project led to a reduction in referrals. The possibility of real-time counter-referral enables continuity of care and longitudinality, in addition to the qualification of assistant doctor in primary care services. As such, the project enables ambulatory care sensitive conditions sensitive to primary care, such as highly prevalent chronic noncommunicable diseases, like diabetes and hypertension, to be correctly managed at this level of care, avoiding unnecessary referrals and overloading of specialized services.

Globally, chronic patients account for 71% of deaths; of these, 53% occur in middle- and low-income countries. Furthermore, 50% of these deaths occur before the age of 70, which is of concern, since these diseases can be prevented and controlled ^20^ ^,^ ^21^. In view of these challenges, the goal is to reduce premature mortality due to noncommunicable diseases by one third through prevention and treatment, promotion of mental health and social well-being ^22^. Among the patients treated by the TeleNordeste project, more than 96% had chronic noncommunicable diseases, and the median age was 50 (36.62) years, that is, within the age range in which approximately 50% of deaths from this cause occur ^23^. In 2019, high blood pressure was the main mortality risk factor in Brazil, followed by smoking and high body mass index. These risk factors are in line with the main diseases reported by patients treated within TeleNordeste project, whereby 60% had cardiovascular diseases, and 51.2% had diabetes and other kidney diseases ^22^.

In addition to the high proportion of chronic patients, the proportion of patients with multimorbidities was also frequent, taking multimorbidity to be the combination of one chronic disease with at least one other disease, biopsychosocial factor or somatic risk factor ^24^. Multimorbidity patients have lower life expectancy and poorer quality of life when compared to patients who have only one disease. Treatment of these patients is complex, since clinical protocols are generally for a single disease, and the application of multiple approaches, even if based on clinical protocols, can lead to interaction between diseases, between treatments, or even between disease and treatment. ^25^. It is important to stress the need for qualified professionals skilled in a comprehensive approach, this being a requirement for primary health care.

Telemedicine helps to reduce waiting times for specialized care. In the United Kingdom, the public National Health Service recommends that, in England, the waiting time for non-urgent consultations with specialists should not exceed 18 weeks. As such, it can be considered that the waiting time experienced by patients treated via the TeleNordeste project - a median of seven days- is adequate ^26^.

Regarding the limitations of this study, there is scarcity of information, such as data on waiting time for consultation with specialists prior to the project being implemented. This would be relevant for making a comparison before and after the intervention, in order to check whether there was a change in this indicator. When determining effectiveness as being the ability to resolve the patient’s complaint at the primary care center, without the need for referral to face-to-face care in the specialty in question, a time frame was established that implies a limitation of the study, since, in the long term, the patient may need to be referred to a higher level of care, considering the progression of their chronic clinical conditions. Even so, in a context of scarcity of resources, postponing the need for these referrals enables optimization of health care network resources, enhancing the expansion of primary care resolutive capacity. Another consideration that must be made is the difficulty of ascertaining, due to the scarcity of records, the financial savings achieved by telecare, as it reduces the patient’s need to travel between their municipality of residence and the main city in their health regions, which, for the most part, is subsidized by the government of the municipality where the patient lives, thus ensuring case management within the local territory for longer. Other limitations found in the TeleNordeste project were related to problems common to telemedicine projects that are independent of joint contributions by health regions, such as internet connection, in addition to distrust of telemedicine by some health professionals, as well as difficulty in collecting data related to socio-environmental impacts that telemedicine interventions can present in the regions in which it operates.

We have presented the results obtained regarding the care of 572 patients who underwent interconsultation through the TeleNordeste project. The data described here indicate that the model has the potential to improve primary health care, as resolutive capacity was higher than the estimate expected to be achieved at this level of care, as well as the potential to contribute to equity from the point of view of the distribution of doctors throughout the territory and to enable professional qualification, due to the improvement that occurs concomitantly with triangulated care with specialists. More robust studies, including from a financial point of view, need to be carried out, so that more evidence about the model can be generated.

## Data Availability

The database used in the research is not available, as other stages of the present study are still underway.
