# Supplementary material for: Specialized medical care in primary care using telemedicine in Northeast Brazil: a descriptive study, Rio Grande do Norte, Brazil, 2022-2023
Source: Epidemiol Serv Saude. 2025 Mar 14;34:e20240256. doi: 10.1590/S2237-96222025v34e20240256.en (PMC11913440; doi:10.1590/S2237-96222025v34e20240256.en)
Supplement: Translation

# Translation: Assistência médica especializada na atenção primária por meio da telemedicina no Nordeste do Brasil: estudo descritivo, Rio Grande do Norte, 2022-2023

**Authors:** Maria Eulália Vinadé Chagas, Gabriel Ricardo Fernandes, Deysi Heck Fernandes, Andressa Dutra Dode, Gabriela Tizianel Aguilar, Tiago Sigal Linhares, Marcilene Batista Costa, Haylla Travassos Caires, Felipe Cezar Cabral, Hilda Maria Rodrigues Moleda Constant, Taís de Campos Moreira

**Affiliations:** 1 Hospital Moinhos de Vento, Responsabilidade Social, Porto Alegre, RS, Brasil; 2 Universidade do Vale do Rio dos Sinos, Escola de Saúde, São Leopoldo, RS, Brasil; 3 Universitat de València, Department of Behavioral Sciences Methodology, Valencia, Comunidad Valenciana, Espanha

**Keywords:** Telemedicina, Doenças Não Transmissíveis, Equidade, Atenção Primária à Saúde, Epidemiologia Descritiva

## Abstract

**Objetivo:**

Descrever um projeto de interconsultas com especialista e equipes de saúde da família aderentes a um projeto de telemedicina no Rio Grande do Norte.

**Métodos:**

Estudo descritivo com avaliação das interconsultas do projeto TeleNordeste. A coleta de dados iniciou-se em novembro de 2022, com as interconsultas de cardiologista, neurologista, psiquiatra e endocrinologista ofertadas para as unidades básicas. Foram inseridos no estudo pacientes maiores de 18 anos. Os dados quantitativos foram descritos como medianas e percentis, os dados qualitativos foram medidos em frequência absoluta e percentual.

**Resultados:**

Foram inseridos 572 pacientes que realizaram 847 interconsultas; 71% eram mulheres, a mediana de idade era de 50 anos, 96,7% dos pacientes tinham doenças crônicas não transmissíveis. A mediana do tempo de espera para interconsulta foi 7 dias. Ao todo, 565 pacientes tiveram sua queixa completamente resolvida e não precisaram ser encaminhados a serviço especializado.

**Conclusão:**

O projeto TeleNordeste levou ao Rio Grande do Norte modalidade de atendimento médico facilitada pela saúde digital, com possibilidade de contato ágil e fácil acesso a cardiologistas, neurologistas, psiquiatras e endocrinologistas na atenção primária à saúde, possibilitando aprimoramento do atendimento e aumento da efetividade em tempo real.

**Aspectos éticos**

A presente pesquisa respeitou os princípios éticos, obtendo os seguintes dados de aprovação:

Comitê de ética em pesquisa: Hospital Moinhos de Vento

Número do parecer: 5.744.857

Data de aprovação: 8/11/2022

Certificado de apresentação de apreciação ética: 63070522.8.0000.5330

Termo de consentimento: Obtido de todos os participantes antes da realização do tele-atendimento.

## Introdução

A adoção de tecnologias de informação na saúde ampliou a diversificação da telemedicina, abrangendo não apenas assistência médica, mas também o *e-learning* e teleconsultorias, como segundas opiniões de especialistas ^1^ ^,^ ^2^. A saúde digital proporciona acesso equiparado ao atendimento disponível em centros urbanos quando ocorre de forma síncrona ^1^ ^,^ ^2^, sendo importante para atingir as metas do terceiro Objetivo Sustentável da Agenda 2030, que enfoca, como caminho necessário à saúde e bem-estar, a cobertura universal, do ponto de vista econômico e de acesso seguro e eficaz à assistência, a medicamentos e a vacinas ^2^ ^,^ ^3^.

No Brasil, as iniquidades em saúde são influenciadas tanto pela extensão territorial quanto pela distribuição da densidade de médicos ^4^. O Nordeste possui 1,93 médico por 1 mil habitantes, sendo a segunda região com densidade entre as mais baixas do país. As múltiplas realidades de acesso à saúde especializada criam vazios assistenciais, os quais são intensificados pela falta de gestão horizontalizada e coordenação de cuidado alinhado nos três níveis de gestão: municipal, estadual e federal ^5^ ^-^ ^7^. A cobertura irregular de saúde por médicos é preocupação maior nas áreas onde o índice de desenvolvimento humano é muito baixo, como no Nordeste do Brasil ^8^. Essa iniquidade, herdada de limitações de políticas regionais explícitas no sistema de saúde ^9^ e da distribuição desarticulada das transferências de financiamento, evidencia o pressuposto da lei do cuidado reverso ^10^, pela existência da disponibilidade de assistência médica inversa às necessidades sociais ^11^. Esta dinâmica reforça a importância de repensar a organização dos serviços de saúde fora da lógica da economia de mercado ^9^ ^,^ ^12^.

Compreender os desafios no acesso à saúde, incluindo a distribuição desigual de médicos e a escassez de recursos humanos para tarefas administrativas, destaca a urgência de projetos que promovam o aprimoramento do Sistema Único de Saúde (SUS) e atendam às demandas específicas de cada região, especialmente na área da atenção primária à saúde. Desta forma, por meio da parceria público-privada, o Programa de Apoio ao Desenvolvimento Institucional do SUS, alinhando-se à Estratégia Global de Saúde Digital 2020-2025, propõe, com cinco hospitais de excelência do Brasil, em conjunto com o Ministério da Saúde e o Conselho Nacional de Secretários de Saúde, desenvolver o projeto “Assistência Médica Especializada na Região Nordeste do Brasil por Meio da Telemedicina - projeto TeleNordeste.” O projeto tem como propósito a prestação de assistência e amparo à gestão na região Nordeste do país. O objetivo deste trabalho é descrever projeto de interconsultas com especialistas e equipes de saúde da família aderentes a projeto de telemedicina no Rio Grande do Norte.

## Métodos

### Delineamento 

Trata-se de estudo descritivo das interconsultas realizadas pelo projeto TeleNordeste entre 30/11/2022 e 30/11/2023, analisando as características demográficas, as condições crônicas dos pacientes atendidos e o acompanhamento dos encaminhamentos realizados para atendimentos presenciais. Os dados foram reportados de acordo com as diretrizes para estudos transversais ^13^.

### Contexo

TeleNordeste é um projeto de assistência e pesquisa, desenvolvido no âmbito do Programa de Apoio ao Desenvolvimento Institucional do SUS, que teve início em novembro de 2022 e tem por objetivo oferecer interconsultas com cardiologista, neurologista, psiquiatra e endocrinologista para Unidades Básicas de Saúde (UBS). Os médicos das especialidades, ficavam alocados no estado do Rio Grande do Sul, localizado na região Sul do Brasil. As UBS estavam situadas em três regiões de saúde das duas macrorregiões do estado do Rio Grande do Norte, situado no Nordeste. Utilizou-se uma plataforma de telemedicina desenvolvida pelo projeto TeleNordeste em que eram realizados o cadastro dos pacientes, os agendamentos, as consultas e no qual o especialista registrava a evolução do paciente. Para garantir a segurança da plataforma e o sigilo da consulta, somente a equipe do projeto TeleNordeste e os médicos e enfermeiros das Equipes de Saúde da Família tinham acesso à ferramenta, com usuários pessoais, recebidos após a realização de treinamento sobre o uso da plataforma e confirmação do termo de aceite do uso e das políticas de privacidade da plataforma. A plataforma de telemedicina passou por avaliação de agentes externos, para verificação dos mecanismos de segurança, sendo aprovada para uso. Não houve o armazenamento de vídeo ou voz dos participantes da interconsulta. Todos os dados pessoais dos participantes das interconsultas foram armazenados na plataforma seguindo os critérios da Lei Geral de Proteção de Dados Pessoais ^14^. 

Os atendimentos foram realizados por videoconferência, de forma triangulada entre o médico especialista, o médico da atenção primária à saúde e o paciente, na modalidade de interconsulta, ou seja, troca de informações e opiniões entre médicos para investigação diagnóstica ou manejo terapêutico e clínico ^15^. A triangulação do atendimento visa fortalecer a diretriz da descentralização do SUS e os atributos da atenção primária à saúde, com a presença do médico do território, mantendo-se assim a coordenação, integralidade e longitudinalidade do cuidado. 

### Participantes

Os pacientes maiores de 18 anos com demandas para as especialidades médicas de cardiologia, endocrinologia, psiquiatria ou neurologia foram elegíveis à interconsulta no projeto TeleNordeste e incluídos na pesquisa por meio de amostragem de conveniência.

A data do atendimento ficava a cargo da agenda do profissional médico da atenção primária à saúde. No dia e horário agendado, o paciente comparecia à UBS, era aplicado o termo de consentimento livre e esclarecido e, então, juntamente com o seu médico de referência, conectava-se com o especialista para a realização do atendimento. A consulta era conduzida com as três partes interagindo ativamente, com o especialista sugerindo condutas ou demandando informações a mais para compreender a integralidade dos casos. Se necessário, o médico especialista focal poderia solicitar ao médico da UBS que realizasse exame físico, ou manobras específicas, oferecendo orientações de como realizá-las, bem como passar mais detalhes de informações da anamnese. Ao final do atendimento, o paciente poderia receber alta, ter nova interconsulta para cuidado continuado agendada ou ser encaminhado para atendimento presencial. Todas as condutas do plano de cuidado eram definidas em conjunto pelos médicos, tendo em vista aspectos socioeconômicos e culturais do paciente, além dos recursos disponíveis via SUS na região (Figura 1).

### Variáveis

O desfecho primário foi cuidado efetivo, considerado quando a queixa principal foi resolvida na interconsulta. As variáveis do estudo incluíram sexo, idade, presença de doenças crônicas, multimorbidade, utilização da agenda e grupo de doenças crônicas não transmissíveis.

**Figura 1:** Processo do paciente no Projeto TeleNordeste desde a indicação para realização de interconsulta até a definição de conduta e análise dos dados do paciente

### Fonte de dados e mensuração

Os dados do estudo foram coletados entre novembro de 2022 e novembro de 2023, por meio de consulta ao prontuário do paciente, e armazenados em planilha eletrônica. Foram inseridos apenas os pacientes que assinaram termo de consentimento livre e esclarecido

As doenças crônicas não transmissíveis foram categorizadas em 12 grupos, conforme o Global Burden of Disease, com exceção do grupo adicional intitulado “outras doenças metabólicas”, elaborado pelos autores ^16^. Este grupo classificou os pacientes que apresentavam alguma síndrome metabólica não relacionada ao diabetes ou problemas renais, dada a alta prevalência da condição nos pacientes atendidos. 

### Métodos estatísticos

Empregou-se estatística descritiva, com cálculo de medianas e percentis para dados contínuos e frequência absoluta e percentual para categóricos.

## Resultados

Foram incluídos no estudo 572 pacientes, que realizaram 847 interconsultas pelo projeto TeleNordeste. A mediana de idade dos pacientes atendidos foi 50 anos (intervalo interquartílico 36;62). As características demográficas estão apresentadas na Tabela 1.

**Tabela 1:** Características demográficas dos pacientes atendidos pelo projeto TeleNordeste. Rio Grande do Norte, 2022-2023 (n=572)

Variável	n (%)
**Sexo**	572 (100)
Feminino	406 (71)
Masculino	166 (29)
**Doença crônica não transmissível**	553 (96,7)
**Multimorbidade**	553 (96,7)

O cuidado foi considerado efetivo em 98,7% (n=565) dos pacientes que tiveram sua queixa principal resolvida na interconsulta, sem a necessidade de encaminhamento para o atendimento presencial na mesma especialidade. Entre os sete pacientes que precisaram ser encaminhados para atendimento presencial, três foram atendimentos cardiológicos, dois endocrinológicos, um psiquiátrico e um neurológico. Os motivos de encaminhamento foram avaliação de cirurgia cardíaca e dois laudos pré-operatórios com risco cirúrgico em cardiologia, diabetes tipo 1 e paciente com complicações macrovasculares em endocrinologia, paciente encaminhado para avaliação presencial de emergência em psiquiatria e avaliação para aplicação de toxina botulínica em neurologia.

Das 847 interconsultas realizadas, 71,8% (n=608) foram de primeira consulta e 28,2% (n=239) para cuidado continuado. A mediana do tempo de espera para interconsulta foi 7 dias considerando-se as quatro especialidades. Para cardiologia, a mediana foi 5 dias; para psiquiatria e neurologia, a mediana foi 7 dias; e para endocrinologia, 10 dias.

A utilização dos horários disponibilizados por especialidade no projeto, entendida como a razão entre o número de interconsultas realizadas e o número de interconsultas agendadas, pode ser visualizada na Tabela 2.

**Tabela 2:** Utilização da agenda por especialidade. Rio Grande do Norte, 2022-2023 (n=572)

Especialidade	Utilização da agenda (%)
Cardiologia	235 (84)
Endocrinologia	281 (75)
Neurologia	117 (80)
Psiquiatria	214 (84)

Foram coletadas as principais doenças crônicas não transmissíveis dos 572 pacientes atendidos. A coleta resultou em 1.149 doenças notificadas, que foram classificadas em 12 grupos, apresentados na Tabela 3.

**Tabela 3:** Número absoluto e frequência das principais doenças crônicas não transmissíveis. Rio Grande do Norte, 2022-2023 (n=1.149)

Grupo de doença	n (%)
Doenças cardiovasculares	364 (60,5)
Diabetes e doença renal	293 (51,2)
Outras doenças metabólicas	182 (31,8)
Doenças mentais	157 (27, 5)
Doenças neurológicas	77 (13, 5)
Doenças musculoesqueléticas	25 (4,4)
Uso de substância psicoativas	20 (3,5)
Doenças de órgãos sensoriais	7 (1,2)
Doenças respiratórias crônicas	6 (1,0)
Doenças digestivas	5 (0,9)
Neoplasias	4 (0,7)
Outras^a^	9 (1,6)

Nota: ^a^Anemia, dor crônica, gota, lúpus, miomatose uterina e paralisia infantil.

## Discussão

A implementação do projeto TeleNordeste levou ao Rio Grande do Norte modalidade de atendimento médico facilitada pela saúde digital, com a possibilidade de contato ágil e de fácil acesso a cardiologistas, neurologistas, psiquiatras e endocrinologistas na atenção primária à saúde, possibilitando aprimoramento do atendimento e aumento da efetividade em tempo real. No TeleNordeste, a medição da efetividade do cuidado foi definida como a capacidade de resolver a queixa sem a necessidade de encaminhamento do paciente para outro nível de atenção. Obteve-se alta efetividade, visto que 565 pacientes atendidos no período tiveram sua queixa completamente resolvida e não precisaram ser encaminhados para serviço especializado.

No Brasil, estima-se que a atenção primária à saúde seja capaz de resolver cerca de 80% dos problemas de saúde da população, e apenas 4% a 9% dos pacientes atendidos são encaminhados para níveis de atenção especializados ^17^ ^,^ ^18^. Observa-se que, na maioria dos casos, os encaminhamentos da atenção básica para serviços de atenção especializada são, em grande parte, determinados pela experiência prévia do médico no manejo de certas doenças, de modo que a demanda por encaminhamentos varia também conforme a especialidade ou expertise do profissional que atua na atenção básica ^19^. Com o projeto TeleNordeste houve redução nos encaminhamentos. A possibilidade de contrarreferência em tempo real possibilita a continuidade do cuidado e longitudinalidade, além da qualificação do profissional assistente na atenção primária. Assim, o projeto possibilita que condições sensíveis à atenção primária, como as doenças crônicas não transmissíveis altamente prevalentes, como diabetes e hipertensão, sejam corretamente manejadas nesse nível de atenção, evitando encaminhamentos desnecessários e sobrecarga dos serviços especializados. 

Globalmente, os pacientes crônicos representam 71% das mortes; destes, 53% estão entre países de média e baixa renda. Além disso, 50% desses óbitos ocorrem antes dos 70 anos, o que é alarmante, uma vez que essas doenças são passíveis de prevenção e controle ^20^ ^,^ ^21^. Frente a esses desafios, foi pactuada meta de redução de um terço da mortalidade prematura por doenças não transmissíveis via prevenção e tratamento, promoção de saúde mental e bem-estar social ^22^. Entre os pacientes atendidos pelo projeto TeleNordeste, mais de 96% tinham doenças crônicas não transmissíveis, e a mediana de idade era de 50 (36,62) anos, ou seja, dentro da faixa etária em que cerca de 50% dos óbitos por essa razão ocorrem ^23^. Em 2019, a pressão arterial elevada foi o principal fator de risco para mortalidade no Brasil, seguida de tabagismo e índice de massa corporal elevado. Esses fatores de risco estão em consonância com as principais doenças relatadas por pacientes atendidos pelo TeleNordeste, em que 60% tinham doenças cardiovasculares, e 51,2%, diabetes e outras doenças renais ^22^.

Além da alta proporção de pacientes crônicos, pacientes com multimorbidades também foi frequente, considerando-se multimorbidade a combinação de uma doença crônica com ao menos uma outra doença, fator biopsicossocial ou fator de risco somático ^24^. Pacientes com multimorbidade tem menor expectativa e qualidade de vida quando comparados a pacientes com uma doença apenas. O tratamento desses pacientes é complexo, uma vez que os protocolos clínicos geralmente são para uma única doença, e a aplicação de múltiplas condutas, ainda que baseada em protocolos clínicos, pode levar à interação entre doenças, entre tratamentos, ou até entre doença e tratamento ^25^. É importante reafirmar as necessidades das competências dos profissionais qualificados para trabalhar na integralidade, a qual se exige na atenção primária.

A telemedicina auxilia em relação ao tempo de espera para o atendimento especializado. O National Health Service, sistema público de saúde do Reino Unido, preconiza que, na Inglaterra, o tempo de espera para consultas não urgentes com especialistas seja de no máximo 18 semanas. Dessa forma, é possível compreender que o tempo de espera experienciado pelos pacientes atendidos no TeleNordeste - mediana de sete dias de espera - está adequado ^26^.

Com relação às limitações deste estudo, temos a escassez de informações, como dados de tempo de espera para consulta com especialistas anteriores à implementação do projeto. Isso seria relevante para realizar um comparativo antes e depois da intervenção, a fim de se verificar se houve alteração nesse indicador. Ao determinarmos a efetividade como a capacidade de resolver a queixa do paciente nas UBS, sem a necessidade de encaminhamento para o atendimento presencial na mesma especialidade, estabeleceu-se marco temporal que implica em limitação do estudo, uma vez que, em longo prazo, o paciente pode necessitar de encaminhamento para outro nível de atenção, considerando-se a evolução de suas condições clínicas crônicas. Ainda assim, em um contexto de escassez de recursos, postergar a necessidade desse encaminhamento permite a otimização dos recursos da rede de atenção à saúde, potencializando a ampliação da resolutividade da atenção primária. Outra consideração que deve ser feita é a dificuldade de constatar, pela escassez de registros, a economia financeira do teleatendimento, por diminuir a necessidade de deslocamento do paciente entre seu município de residência e as cidades sedes das regiões de saúde, que, em sua maioria, é subsidiada pelo próprio município de residência do paciente, garantindo o manejo dentro do território por mais tempo. Outras limitações encontradas no projeto TeleNordeste foram relacionadas a problemas comuns a projetos de telemedicina que independem de contrapartida das regiões, como conexão com a internet, desconfiança por parte de alguns profissionais na telemedicina, bem como dificuldade de coletar dados relativos a impactos socioambientais que a intervenção por telemedicina pode apresentar nas regiões em que atua.

Apresentamos os resultados obtidos a partir do atendimento de 572 pacientes que realizaram interconsulta pelo projeto TeleNordeste. Os dados aqui descritos indicam que o modelo tem potencial de aprimorar a atenção primária à saúde, visto que a resolutividade foi superior à estimativa que se espera atingir nesse nível de atenção, de contribuir com a equidade do ponto de vista de distribuição médica pelo território e de possibilitar a qualificação do profissional, devido ao aprimoramento que ocorre concomitante aos atendimentos triangulados com os especialistas. Estudos mais robustos, inclusive do ponto de vista financeiro, precisam ser realizados, para que mais evidências sobre o modelo sejam geradas.

## Data Availability

O banco de dados utilizado na pesquisa não está disponível, visto que outras etapas do presente estudo seguem em execução.
